# Phylogenetic Groups and Antimicrobial Susceptibility Patterns of* Escherichia coli* from Healthy Chicken in Eastern and Central Uganda

**DOI:** 10.1155/2018/9126467

**Published:** 2018-08-07

**Authors:** Winston Kabiswa, Ann Nanteza, Gabriel Tumwine, Samuel Majalija

**Affiliations:** ^1^Department of Biosecurity, Ecosystem and Veterinary Public Health, College of Veterinary Medicine, Animal Resources and Biosecurity, Makerere University, P.O. Box 7062, Kampala, Uganda; ^2^Department of Biomolecular Resources and Biolab Sciences, College of Veterinary Medicine, Animal Resources and Biosecurity, Makerere University, P.O. Box 7062, Kampala, Uganda

## Abstract

Antimicrobial resistance is an emerging problem in both humans and animals due to misuse and excessive use of drugs. Resistance in commensal* E. coli* isolates can be used to predict emergence of resistance in other gut microflora. The aim of this study is to determine the phylogenetic groups and antimicrobial resistance patterns of* E. coli* from healthy chickens in Uganda. The phylogenetic grouping of 120 fecal* E. coli* isolates from eastern and central Uganda was derived using the triplex PCR assay and their susceptibility patterns determined by agar disc diffusion method to 5 antimicrobial drugs. Most* E. coli* is segregated into phylogenetic group A comprising 84%, while 12% and 4% were in groups D and B1, respectively. Similarly most* E. coli* from central (87%) and eastern Uganda (82%) belonged to group A. Overall, 85 (70%) of* E*.* coli *were resistant to antimicrobial drugs, of which 72/101 (70%) are in PG A, 10 of 14 (71.4%) in PG D, and 3 of 5 (60%) in PG B1. Significantly, most of the isolates in PG A from both central (66.7%) and (60.6%) eastern Uganda were resistant to one antimicrobial. Resistance to tetracycline alone or in combination with other drugs for central and eastern Uganda in PG A is 51% and 55%, respectively. Multidrug resistance to tetracycline and ciprofloxacin or nalidixic acid was 10% and 18% in isolates from central and 10% and 12% in isolates from eastern region, respectively. Phylogenetic group A accounts for most of the* E. coli* in chicken from Uganda. No difference in the resistance rates between the phylogenetic groups of* E. coli* has been observed. The high prevalence of resistant* E. coli* strains from different phylogenetic groups in healthy chickens suggests antimicrobial drug selection pressure due to excessive drug in the rearing layer chickens.

## 1. Background

*E. coli* is a commensal organism within the gastrointestinal tract of warm blooded animals. In the recent past, strains known to cause illness in animals and humans have emerged [1, 2]. In chicken, pathogenic strains cause respiratory infections, pericarditis, septicemia [3], and colibacillosis [4, 5].* E. coli* is a ubiquitous organism. Its adaptation to the diverse ecological niches including the intestinal and extraintestinal sites, as well as sites outside the host [6], is aided by the flexibility of the genome; exchange, retention, and/or loss of accessory genetic elements takes place [6, 7].

Antimicrobial drugs are commonly used in Uganda and other countries to prevent and treat diseases and as growth promoters in poultry proudction [8]. Equivocally, indiscriminate drug use exerts high antibiotic selection pressure on chicken gut coliforms which leads to emergence of antibiotic-resistant* E. coli* phenotypes [9, 10] and shed in faeces [11]. The presence of resistant* E. coli* is a strong predictive indicator for emergence of resistance in other organisms (pathogenic and nonpathogenic) within gastrointestinal tract of the chicken [11].

The genetic background of* E. coli* reflects its evolutionary lineage [12] and strains that evolved along distinct lineages carry specific genetic backgrounds [13]. According to Clermont et al. [14],* E. coli* segregate into the four major phylogenetic groups, namely, A, B1, B2, and D. The commensal strains belong mainly to phylogenetic groups A and B1 [15]. Strains with phylogenetic groups B2 and D carry virulence determinants [15, 16]. Phylogenetic studies are important to improve the understanding of the lineages of* E. coli* population; however, such information is not available for* E. coli* strains in chicken from Uganda. Therefore the study investigates the genetic background and the occurrence of antimicrobial resistance of* E. coli* from healthy chickens in Uganda.

## 2. Materials and Methods

### 2.1. Bacterial Isolates

Previously archived* E.coli* strains from healthy chicken in central and eastern Uganda collected from May 2010 to September 2011 were used. These had been stored in microbiology laboratory of College of Veterinary Medicine, Animal Resources and Biosecurity, Makerere University. One hundred twenty isolates were identified as* E. coli* by the standard biochemical tests [17].

### 2.2. Genomic DNA Was Extracted

Bacterial genomic DNA was extracted using the rapid boiling method described by Wang et al. (2010). A single colony of* E. coli* was grown overnight on Brain Heart Agar (Oxoid™) for 24 hours at 37°C. A loop-full of colonies was suspended in 0.5 ml of double distilled sterile water; cells were lysed at 95°C for 10 minutes. After cooling to room temperature, the suspension was centrifuged at 12,000 rpm for 3 minutes to remove cell debris. The supernatant containing template DNA was used for PCR.

### 2.3. Phylogenetic Typing

Triplex PCR-based method as described by Clermont et al. [14] was used. All strains were assigned to 1 of the 4 major* E. coli* phylogenetic groups (A, B1, B2, and D). The* E. coli* K-12 (phylogroup A), STEC O111 (phylogroup B1), and O157:H7 (phylogroup D) were used as positive controls.

### 2.4. Antimicrobial Susceptibility Test

Antimicrobial susceptibility testing was performed on* E. coli* isolates using Kirby-Bauer disc diffusion method [18] as recommended by CLSI [19]. It was carried out on Mueller-Hinton agar to chloramphenicol 30 *μ*g, nalidixic acid 30 *μ*g, ciprofloxacin 5 *μ*g, gentamicin 10 *μ*g, and tetracycline 30 *μ*g (Oxoid), which are frequently used in poultry production. The mean zone of inhibition of three replicates was used to determine the susceptibility of the isolates [19].* Escherichia coli* ATCC 25922 was used as control strain.

## 3. Results

### 3.1. Phylogenetic Distribution of* E. coli* from Chicken in Eastern and Central Uganda

**Figure 1:**
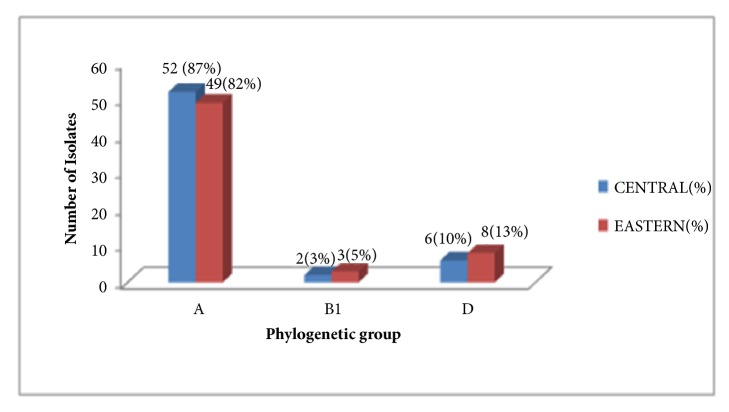
Phylogenetic distribution of* E.coli* from chicken in Eastern and Central Uganda.

**Figure 2:**
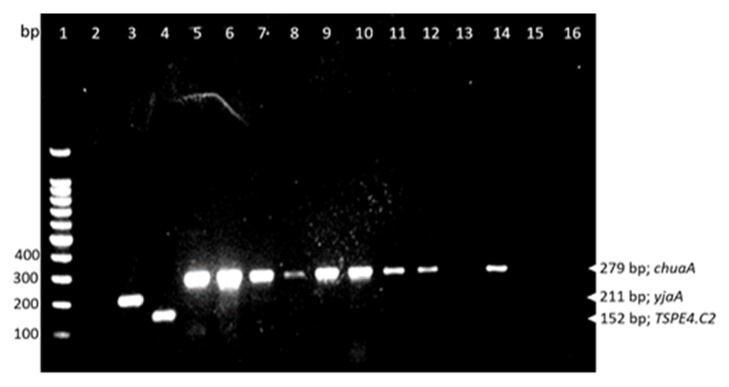
PGs of selected* E.coli* isolates from chicken in Uganda. Lane 1, hyper ladder (100bp DNA ladder, Promega Madison, USA); Lane 2, negative control, no DNA template); Lane 3,* E. coli* K-12 (PG, A); Lane 4, STEC 0111 (PG, B1); Lane 5, 0157:H7 (PG, D); Lanes 6-12 and 14,* E.coli* with PG-D; Lanes 13,15, and 16,* E. coli* with PG-A.

Most E. coli is segregated into phylogenetic group A comprising 84% (101 of 120), while 12% (14) and 45(5) of the isolates were in groups D and B1, respectively. Similarly a majority of* E. coli* from central Uganda, 52 (87%), was segregated into phylogenetic group A, 6 (10%) in D, and 2 (3.3%) in B1; whereas, for eastern Uganda, 49 (82%) were segregated in A, 3 (5%) in B1, and 8 (13%) in D. None of the* E. coli* in phylogenetic group B2 was detected (Figures 1 and 2).

### 3.2. Antibiotic Resistance Profiles of* E.coli *Isolates in relation to Phylogenetic Groups

**Figure 3:**
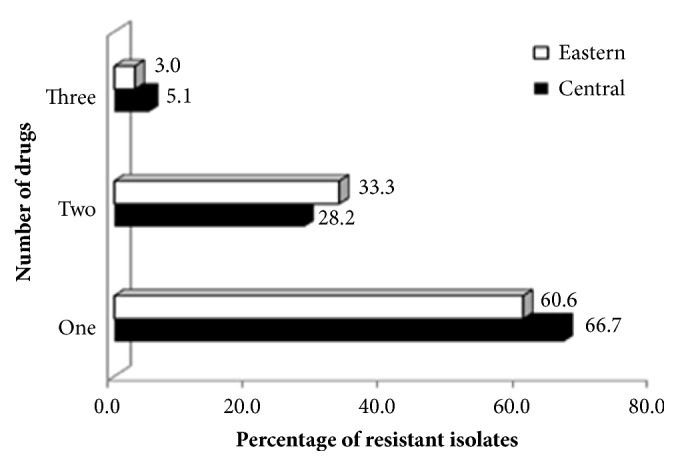
Resistant* E. coli* isolates to one or multiple antimicrobial drugs.

Overall, 85 of 120 (70%)* E. coli *isolates were resistant to antimicrobial drugs. With respect to phylogenetic grouping, 72/101 (70%) in PG A, 10 of 14 (71.4%) in PG D, and 3 of 5 (60%) in PG B1 were resistant. Most of the isolates in PG A from both central (66.7%) and (60.6%) eastern Uganda were resistant to one antimicrobial drug compared to being resistant to two or more antimicrobials (Figure 3).

As regards phylogenetic group A, of the 72* E. coli* that are resistant, 86% (62/72) were resistant to tetracycline, 22%, 21%, and 8% were resistant to ciprofloxacin, nalidixic acid, and chloramphenicol, respectively. A total of 39 of 49 (80%) and 33 of 52 (63%)* E. coli* isolates from central and eastern Uganda, respectively, were resistant. Of these, 20 of 39 (51%) and 18 of 33 (55%) from central and eastern Uganda, respectively, showed resistance to tetracycline alone or in combination with other drugs (Table 1). Multidrug resistance to tetracycline and ciprofloxacin or nalidixic acid was 10% and 18% in isolates from central and 10% and 12% in isolates from eastern region, respectively. Five and 3% of the isolates from central and eastern regions, respectively, were resistant to a combination of tetracycline, ciprofloxacin, and nalidixic acid (Table 1).

Resistance was second highest for ciprofloxacin in the central region with 20% resistant to ciprofloxacin alone (5%), in combination with tetracycline (10%), or with other two drugs (5%). Similarly, 24% from eastern region were resistant to ciprofloxacin, of which majority (18%) were also resistant to tetracycline. Resistance to nalidixic acid alone or in combination with other drugs was observed in 24% and 17.6% of isolates from eastern and central regions, respectively. Significantly, 15.5% of isolates from central regions were resistant to chloramphenicol and none from the eastern region (Table 1).

Of the 10 resistant isolates in PG D, 4 (40%) from central Uganda were all resistant to tetracycline, while two were in addition resistant to chloramphenicol or nalidixic acid. Similarly all 6 (60%) of isolates from eastern region were resistant to tetracycline and two of these were also resistant to chloramphenicol and ciprofloxacin or nalidixic acid, while in PG 3 of 5 isolates in PG B1 were also resistant to ciprofloxacin (1 isolate) from central and tetracycline alone or in combination with chloramphenicol for each isolate from eastern Uganda (Table 1).

## 4. Discussion

Phylogenetic grouping was determined using a simple, quick, reproducible assay [14] that is most suitable for resources limited laboratories. The different strains of* E. coli *were predominantly separated into phylogenetic group A and then D, but none in B2. This comparable phylogenetic distribution of* E. coli* in chicken in both central and eastern regions of Uganda shows that* E. coli *within the chicken population evolved from a recent common ancestry and have established mutual coexistence over millions of years. Phylogenetic group A isolates were predominate in our study which considered healthy chicken. More often, phylogenetic group A* E. coli *isolates are commensal organisms, associated with healthy chicken, and rarely cause disease [20, 21]. Our findings were in agreement with other workers [21, 22]. Conversely, in birds with colibacillosis a majority of* E. coli* belongs to phylogenetic groups B2 [23]. Similarly, the proportion of phylogenetic group B1* E. coli* isolates was the least in strains from chicken in this study. The majority of* E. coli* from poultry in this group are usually Enteropathogenic* E. coli* isolates [20, 21]. Phylogenetic group B2 was absent as expected because* E. coli* in this group are virulent strains of* E. coli* that usually cause infections in chicken [23]. Phylogenetic group D was observed as the second most isolated group of* E. coli *from chicken in Uganda. However, it must be noted that the phylotyping method used in [14] could not distinguish between* E. coli *isolates in groups D and E [24], the latter being associated with severe illness in humans [24]. Thus, there is a need for future studies to confirm the absence or presence of* E.coli* in group E from chicken in central and eastern Uganda.

In this study we examined antimicrobial resistance in commensal* E. coli* isolates from healthy layer chickens from central and eastern Uganda. More than 70% of the isolates were resistant, showing that high prevalence of antimicrobial resistant bacteria in chicken in Uganda is widespread due to misuse of antimicrobials during rearing. Evidently, antibiotic drugs are readily available and administered by farmers without prescription [8, 25], factors that promote the emergence of resistance antibiotic in Uganda. The high level of* E. coli* resistance in chicken is a public health concern, as this organism has a high propensity to disseminate antimicrobial resistance genes to intestinal bacteria in the humans [26, 27]. This may also be an indicator of emerging resistance in other gut microflora within the chicken population.

The isolates were susceptible to gentamicin but more resistant to tetracycline, ciprofloxacin, nalidixic acid, and chloramphenicol. The highest resistance to tetracycline was likely due to extensive use of this drug during chicken rearing for preventive and curative purposes. Tetracycline resistance is easily promoted within* E. coli* population and among other gut microflora because tetracycline resistance genes are located on mobile genetic elements [27, 28]. No resistance was observed to gentamycin, since only injectable formulations are available and rarely used in chicken in Uganda. Similarly, formulations of chloramphenicol are no longer available for use in chicken and hence minimal resistance to this drug. However, the observed resistance can be due to coselection of chloramphenicol resistance by sulphonamides and streptomycin use [29], which are extensively used. Also horizontal transfer of genes from sources like water contaminated with human sewage may be another contributing factor [30]. We detected isolates resistant to more than two drugs; however, this was less frequent compared to other findings where most isolates were resistant to tetracycline, ciprofloxacin, and chloramphenicol [31].

Since the genome of* E. coli* is known to frequently exchange genetic elements including resistance genes [7] we attempted to link the antibiotic resistance pattern to the phylogenetic background. Our results, however, do not show any differences in antibiotic resistance of the strains in the different phylogenetic groups which is in agreement with other workers [24]. Conversely, other studies reported association between phylogenetic group B2 and quinolone-susceptible isolates [32, 33] whereas quinolone-resistant isolates were associated with group A in human isolates [32].

## 5. Conclusion

Phylogenetic group A accounts for most of the* E. coli* in chicken from Uganda. No difference in the resistance rates between the phylogenetic groups of* E. coli* has been observed. The high prevalence of resistant* E. coli* strains from different phylogenetic groups in healthy chickens suggests antimicrobial drug selection pressure due to excessive use in the rearing layer chickens. Rational use of antibiotics may reduce the chances of developing antibiotic-resistant* E. coli* in chickens from Uganda.

## Figures and Tables

**Table 1:** Antibiotic resistance profiles of *E. coli *isolates in phylogenetic groups A and D from central and eastern Uganda.

Number and percentage [ ] of resistant *E. coli* isolates in phylogenetic groups A and D				
Antimicrobial resistance profile	Phylogenetic group A		Phylogenetic group D	

	Central	Eastern	Central	Eastern
	N= 49	N=52	N=6	N=8
T	20 [51.3]	18 [55]	2 [50]	1 [16.7]
C	3 [7.7]	0.0	0	1 [16.7]
CIP	2 [5]	1 [3]	0	1 [16.7]
NA	1 [2.6]	2 [6]	0	0
T+NA	4 [10]	4 [12]	1 [25]	1 [16.7]
T+CIP	4 [10]	6 [18]	0	0
T+C	3 [7.7]	0.0	1 [25]	0
NA+C	0.0	1 [3]	0	0
T+NA+CIP	2 [5]	1 [3]	0	
T+NA+C	0	0	0	1[16.7]
T+CIP+C	0	0	0	1[16.7]
**Total number and **%**age of resistance **	39 [65]	33 [55]	4 [66.7]	6 [75]

T, tetracycline; C, chloramphenicol; CIP, ciprofloxacin; NA, nalidixic acid

## Data Availability

Data is available from the corresponding author on reasonable request.
